# The Hybridge Technique: A Combined Technique of Suture Bridge and Biceps Tenodesis and Lateral Corner of Rotator Cuff Interval Stabilization for an Arthroscopic Rotator Cuff Repair

**DOI:** 10.1002/atn2.70218

**Published:** 2026-08-13

**Authors:** Fei Dai, Ming Xiang

**Affiliations:** ^1^ Department of Upper Limb Sichuan Provincial Orthopaedic Hospital Chengdu China

## Abstract

A rotator cuff (RC) tear is often closely associated with lesions occurring in the long head of the biceps tendon, typically presenting as pulley structural impairment, long head of the biceps tendon instability, and tears at the lateral corner of the RC interval. At present, there are many surgical methods for the injury of the supraspinatus and biceps tendon, but there are many problems such as the number of anchors, the complexity of surgical procedures, and less attention is paid to the lateral corner of the RC interval and the pulley structure. Therefore, we describe a hybrid technique, which uses fewer anchors to repair the supraspinatus and infraspinatus and biceps tenodesis and repair the lateral corner of the RC interval at the same time.

## Graphical abstract

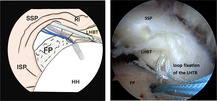

**VIDEO 1:** Right shoulder with lateral decubitus position. Subacromial Posterolateral viewing portal. First, the routine posterior portal is established to introduce the scope. The integrity of the biceps tendon and rotator cuff (RC) is examined to evaluate the stability of the biceps tendon and the extent of RC tear. The anterosuperior portal is established routinely. And then, turn to the subacromial space, and perform debridement. Release the RC interval, coracoid humeral ligament, and retracted supraspinatus and infraspinatus tendons in sequence from anterior to posterior. Insert 2 first‐row anchors, and after loop fixation of the LHTB with 1 set of sutures of the anterior anchor, 2 sutures are sequentially passed through the anterior edge of the supraspinatus and the lateral corner of the RC interval. The other set of sutures of the anterior anchor is threaded together through the supraspinatus tendon to prepare for double‐pulley tying. One set of sutures of the posterior anchor passes through the infraspinatus tendon to prepare for the double‐pulley tying, while the other set of sutures is prepared for side‐to‐side repairing according to the type of RCT. After the distribution of sutures is completed, tie the sutures from anterior to posterior in sequence to complete biceps tenodesis, lateral corner of rotator cuff interval stabilization, and double‐pulley tying and side‐to‐side fixation. According to the size of the rotator cuff tear, use 1 to 2 knotless second‐row anchors to strengthen the fixation of the supraspinatus and infraspinatus tendons. Video content can be viewed at https://doi.org/10.1002/atn2.70218.

Rotator cuff tear (RCT) is often associated with the lesion of the long head of the biceps tendon (LHBT).^1^, ^2^, ^3^ According to a number of studies, LHBT and RCT have a high incidence of complications, such as retears, chronic pain, and functional limitations.^4^, ^5^ At present, there are many clinical techniques and strategies for biceps tendon fixation and rotator cuff (RC) repair, but most of them need to cut the LHBT from the proximal end. It is not clear whether the destruction of the proximal continuity of the LHBT will affect the stability of the shoulder.^6^, ^7^, ^8^ In addition, these techniques do not involve the repair of the lateral corner of the RC interval, so more than 4 or more anchors need to be placed to complete the repair of the supraspinatus and infraspinatus tendons, biceps tenodesis, and RC interval repair.

This technical note describes a hybrid fixation technique, which combines the double‐row suture bridge, biceps tenodesis, and lateral corner of the RC interval repair, using 2 first‐row anchors and 1 or 2 knotless second‐row anchors to repair a large superior cuff tear, while completing the biceps tenodesis and lateral corner of the RC interval repair.

This technique is indicated in medium to large superior cuff tear with LHBT lesion. Contraindications for this technique include partial RCT or small RCT, irreparable massive RCT, and LHBT rupture (Table 1).

**TABLE 1:** Indications and Contraindications

Indications
• Medium to large superior cuff tear with LHBT lesion

Contraindications
• Partial rotator cuff tear or small rotator cuff tear
• Irreparable massive rotator cuff tear
• LHBT rupture

LHBT, long head of the biceps tendon.

## SURGICAL PROCEDURE

The operation is illustrated in Video 1. This hybridge technique is a combined technique of suture bridge and biceps tenodesis and lateral corner of the RC interval stabilization. Table 2 explains the tips, pitfalls, and key points of this technique. The advantages and disadvantages of the technique are listed in Table 3.

**TABLE 2:** Pearls and Pitfalls

Pearls
• Adequate debridement and tendon release are necessary because tendon adhesion and retraction often occur in medium to large rotator cuff tears
• Identify the type of rotator cuff tear and try to reduction to the footprint area
• Place the first row of anchors close to the outside of the bicipital groove
• Reasonably distribute the suture according to the type of rotator cuff tear, including double pulley and side‐to‐side fixation
• Conventional tying from anterior to posterior

Pitfalls
• For elderly people, due to poor bone quality, there is a risk of failure with the second‐row anchor. It is recommended to place the anchor as close to the bicipital groove as possible
• When performing biceps tenodesis, we should pay attention to the tension of biceps tendon and try to fix it in situ

**TABLE 3:** Advantages and Disadvantages

Advantages
• Increasing the coverage area of the rotator cuff footprint and promoting cable healing
• The LHBT is fully preserved, which can continue to exert the biomechanical effect of LHBT in pressing the humeral head and stabilizing the shoulder
• Less anchor, less cost, less wasteful
• Lateral corner of rotator cuff interval stabilization

Disadvantages
• Knot tying is needed, which may affect the blood supply of the rotator cuff
• The sutures need to be distributed according to the type of rotator cuff tear, and the learning curve is relatively long

LHBT, long head of the biceps tendon.

### Preoperative Patient Positioning

After general anesthesia and interscalene block, the patient is placed in the lateral position. Abduct the affected shoulder by 40° through the abduction brace, and carry out appropriate forward flexion traction.

### Portal Establishment and Arthroscopic Examination

The routine posterior portal is established to introduce the scope. The integrity of the biceps tendon and RC is examined to evaluate the stability of the biceps tendon and the extent of RCT. The anterosuperior portal is established routinely.

### Debridement and Release of RC

Establish lateral and anterior‐lateral portals, turn to the subacromial space, and perform debridement. Release the RC interval, coracoid humeral ligament, and retracted supraspinatus and infraspinatus tendons in sequence from anterior to posterior. Identify the type of RCT and try to reduce the footprint area.

### Anchor Insertion and Tendon Suturing

**FIGURE 1:**
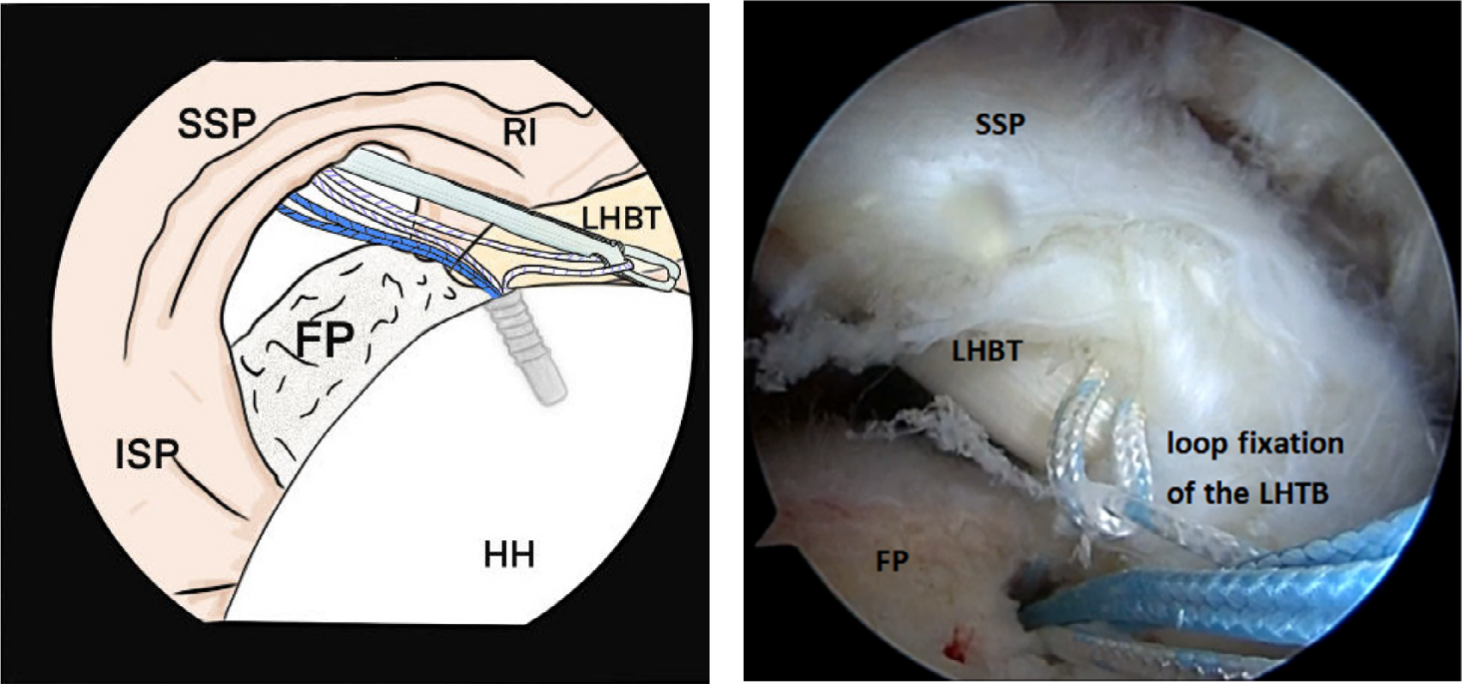
Right shoulder with lateral decubitus position. Subacromial Posterolateral viewing portal. The anterior anchor of the first row should be inserted as close to the bicipital groove as possible. The arthroscopic image shows a subacromial view, with 1 set of sutures of the anterior anchor to perform loop fixation of the LHBT. (FP, footprint; HH, humeral head; ISP, infraspinatus; LHBT, long head of the biceps tendon; RI, rotator cuff interval; SSP, supraspinatus.)

Insert the first row of anchors (FOOTPRINT Ultra PK, Smith & Nephew, Mansfield, MA) through the anterior‐lateral auxiliary portal, with the anterior anchor placed as close as possible to the bicipital groove and the posterior anchor placed along the posterior edge of the tear (Figure 1). It should be noted that special attention should be paid to the direction of anchor placement, and the anchor should be placed in subchondral bone with better bone quality as much as possible to increase anchor stability.

**FIGURE 2:**
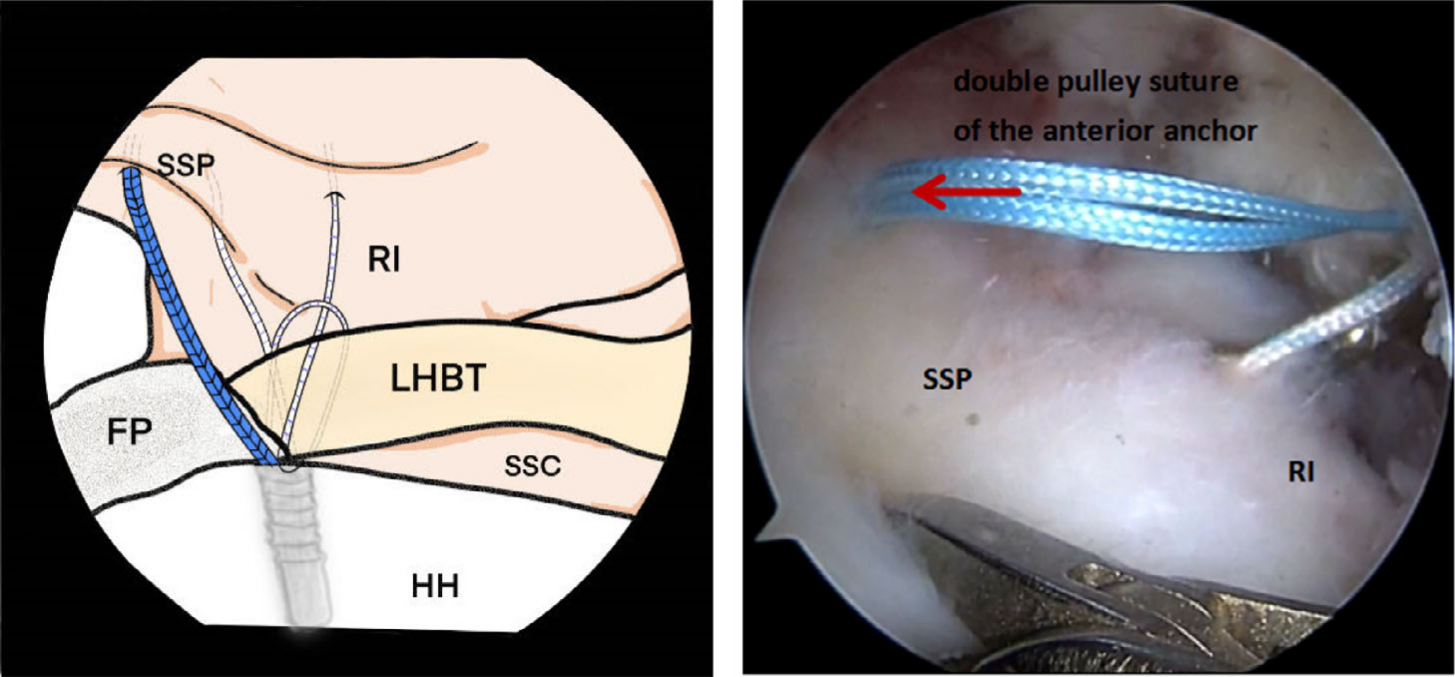
Right shoulder with lateral decubitus position. Subacromial Posterolateral viewing portal. Arthroscopic glenohumeral viewing. After loop fixation of the LHTB with 1 set of sutures of the anterior anchor, 2 sutures are sequentially passed through the anterior edge of the supraspinatus and the lateral corner of the rotator cuff interval. The other set of sutures of the anterior anchor is threaded together through the supraspinatus tendon to prepare for double‐pulley tying. The arthroscopic image shows the subacromial view. After fixing the LHBT, the white suture passes through the anterior edge of the SSP and the lateral corner of the RI, and the blue suture passes through the SSP to prepare for double‐pulley tying (red arrow). (FP, footprint; HH, humeral head; LHBT, long head of the biceps tendon; RI, rotator cuff interval; SSC, subscapularis; SSP, supraspinatus.)

**FIGURE 3:**
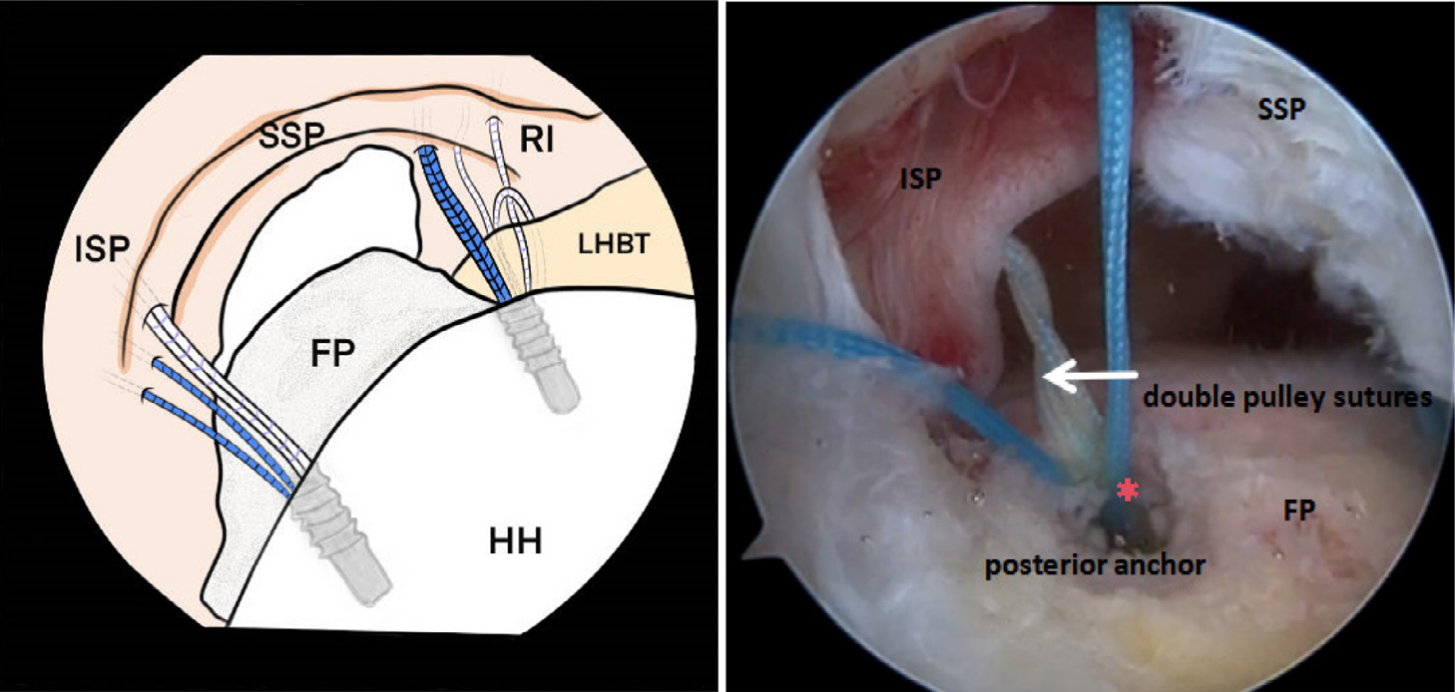
Right shoulder with lateral decubitus position. Subacromial Posterolateral viewing portal. Arthroscopic glenohumeral viewing. One set of sutures of the posterior anchor passes through the infraspinatus tendon to prepare for the double‐pulley tying, while the other set of sutures is prepared for side‐to‐side repairing according to the type of RC tear. The arthroscopic image shows the subacromial view. The white suture (white arrow) of the posterior anchor (red asterisk) passes through the ISP to prepare for double‐pulley tying, and the blue suture passes through the ISP to prepare for side‐to‐side suturing. (FP, footprint; HH, humeral head; ISP, infraspinatus; LHBT, long head of the biceps tendon; RC, rotator cuff; RI, rotator cuff interval; SSP, supraspinatus.)

After loop fixation of the LHTB with 1 set of sutures of the anterior anchor, 2 sutures are sequentially passed through the anterior edge of the supraspinatus and the lateral corner of the RC interval (Figure 2). The other set of sutures of the anterior anchor is threaded together through the supraspinatus tendon to prepare for double‐pulley tying (Figure 3).

**FIGURE 4:**
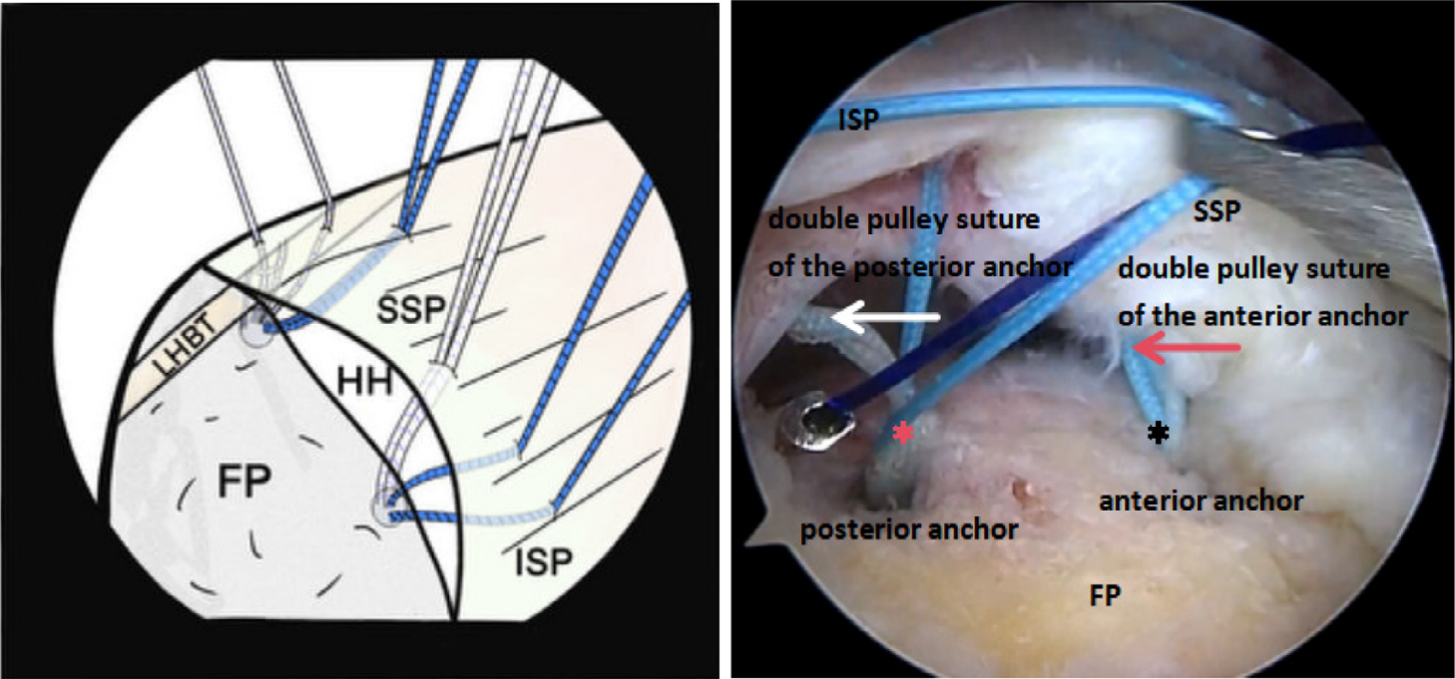
Right shoulder with lateral decubitus position. Subacromial Posterolateral viewing portal. The sutures of the anterior anchor pass through the LHBT and the supraspinatus tendon, respectively. One set of sutures of the posterior anchor passes through the infraspinatus tendon to prepare for the double‐pulley tying, while the other set of sutures is prepared for side‐to‐side repairing according to the type of RC tear. The arthroscopic image shows the distribution of anchors and sutures from the subacromial perspective: the blue suture (red arrow) of the anterior anchor (black asterisk) passes through the SSP to prepare for double‐pulley tying. The white suture (white arrow) of the posterior anchor (red asterisk) passes through the ISP to prepare for double‐pulley tying, and the blue suture passes through the ISP to prepare for side‐to‐side fixation according to the type of RC tear. (FP, footprint; HH, humeral head; ISP, infraspinatus; LHBT, long head of the biceps tendon; RC, rotator cuff; SSP, supraspinatus.)

One set of sutures of the posterior anchor passes through the infraspinatus tendon to prepare for the double‐pulley tying, while the other set of sutures is prepared for side‐to‐side repairing according to the type of RCT (Figure 4).

### Tying and Second‐Row Anchor Insertion

**FIGURE 5:**
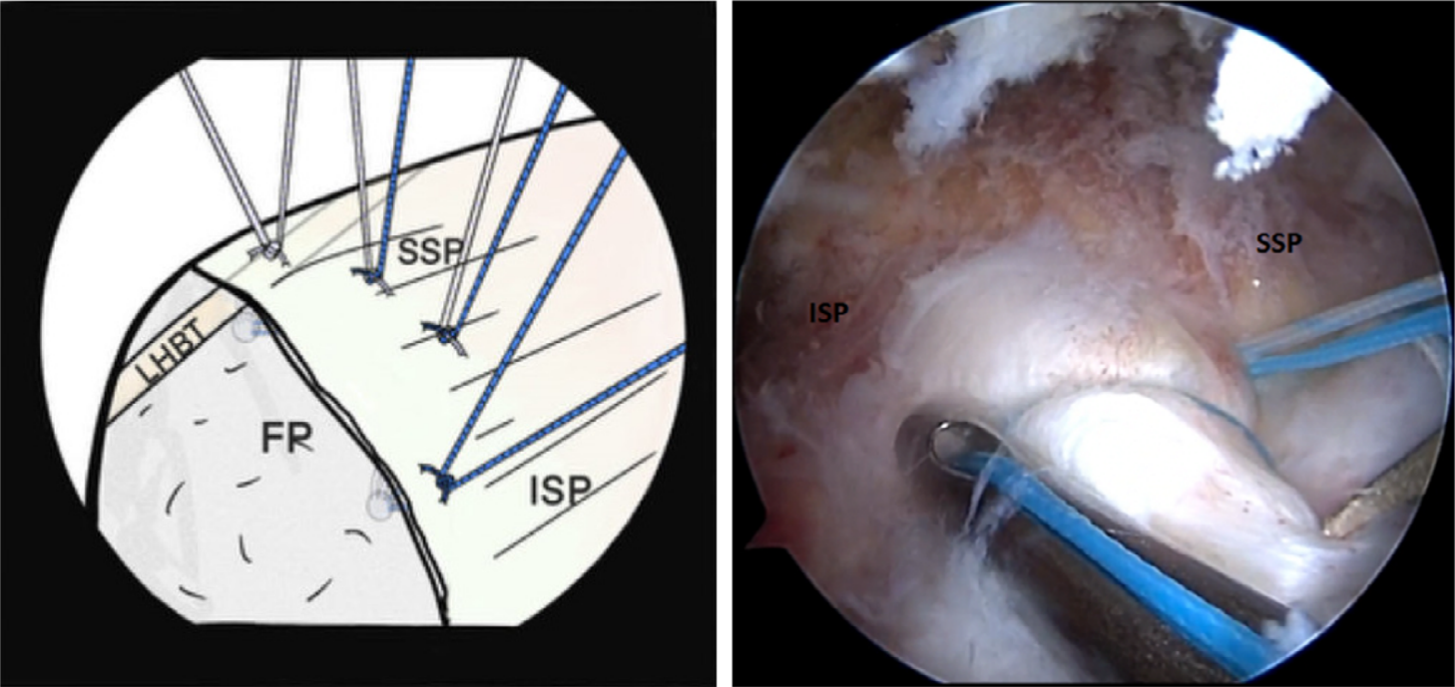
Right shoulder with lateral decubitus position. Subacromial Posterolateral viewing portal. Tying the sutures from anterior to posterior in sequence to complete biceps tenodesis, lateral corner of rotator cuff interval stabilization, and double‐pulley tying and side‐to‐side fixation. The arthroscopic image shows a subacromial view. Double‐pulley tying is being completed. Two sets of sutures (the blue suture of the anterior anchor and the white suture of the posterior anchor) are knotted separately. (FP, footprint; ISP, infraspinatus; LHBT, long head of the biceps tendon; SSP, supraspinatus.)

After the distribution of sutures is completed, tie the sutures from anterior to posterior in sequence to complete biceps tenodesis, lateral corner of the RC interval stabilization, and double‐pulley tying and side‐to‐side fixation (Figure 5).

**FIGURE 6:**
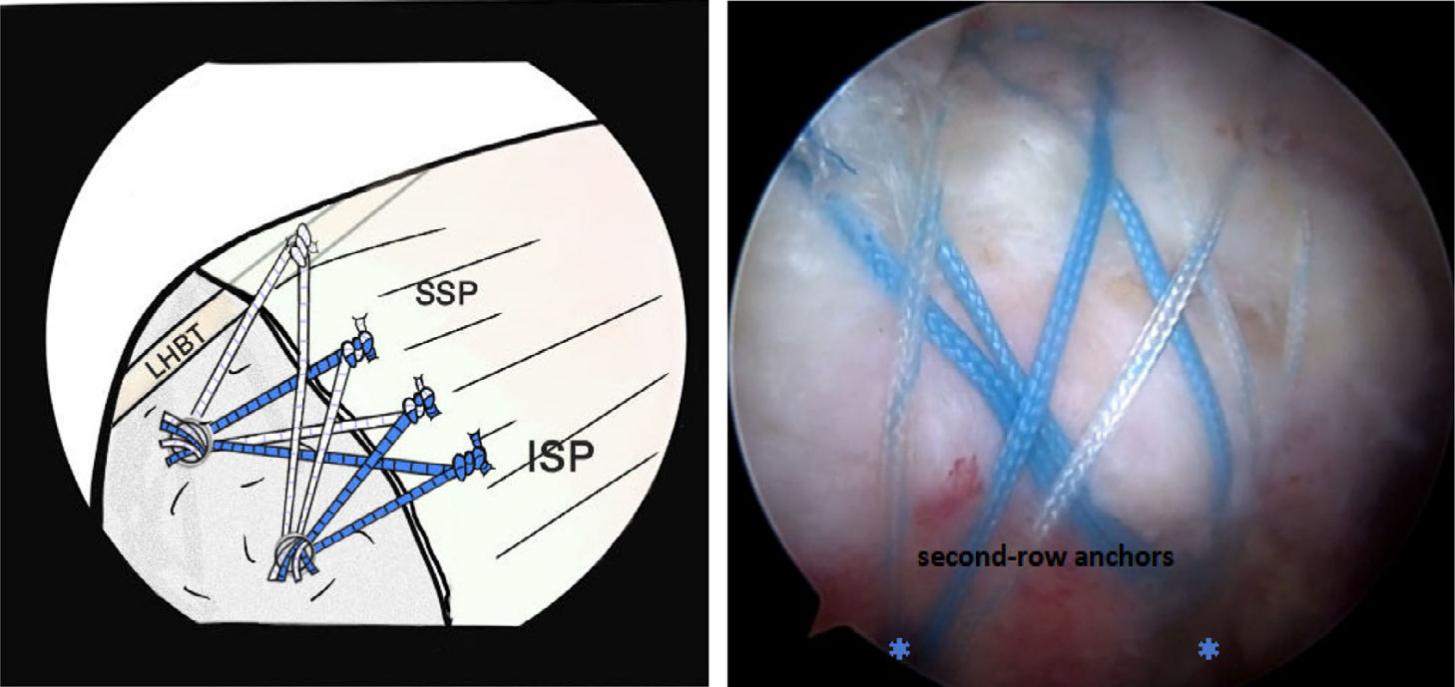
Right shoulder with lateral decubitus position. Subacromial Posterolateral viewing portal. According to the size of the rotator cuff tear, use 1 to 2 knotless second‐row anchors to strengthen the fixation of the supraspinatus and infraspinatus tendons. The arthroscopic image shows a subacromial view. Two second‐row anchors (blue asterisks) strengthen the fixation, increase the contact area between the tendon and the footprints, and provide protection for the first‐row anchors. (ISP, infraspinatus; LHBT, long head of the biceps tendon; SSP, supraspinatus.)

According to the size of the RCT, use 1 to 2 knotless second‐row anchors (REJOIN InLoc PEEK, REJOIN Medical Devices Co., Ltd., Hangzhou, China) to strengthen the fixation of the supraspinatus and infraspinatus tendons. For elderly people, due to poor bone quality, there is a risk of failure with the second‐row anchor. It is recommended to place the anchor as close to the bicipital groove as possible (Figure 6).

### Rehabilitation

The affected shoulder is strictly immobilized with a sling for 4 to 6 weeks after surgery. Passive rehabilitation with elevation and external rotation is allowed at 4 weeks. Passive internal rotation rehabilitation can begin after 8 weeks. At 12 weeks, active exercise can begin gradually. Six months after the surgery, all sports activities are allowed.

## DISCUSSION

For RCTs with combined biceps tendon lesions, few studies support conservative treatment for biceps tendon lesions, but most studies still support more aggressive treatment options, including biceps tenotomy and tenodesis, to alleviate the pain of the shoulder.^9^, ^10^, ^11^ Due to the lower incidence of Popeye malformation and the risk of spastic pain after biceps tenodesis, shoulder function has been better improved in terms of Constant score and Simple Shoulder Test score. More and more surgeons prefer biceps tenodesis, despite its disadvantages of a more difficult operation and longer operation and recovery time.^12^ At present, there are many techniques and strategies for biceps tenodesis, but most of these techniques require cutting the LHBT from the proximal end, and their advantages and disadvantages are still unclear.^4^, ^6^, ^8^ This technique performs biceps tenodesis while repairing the supraspinatus, which can fully preserve LHBT while relieving shoulder pain and avoiding Popeye malformation. In addition, this technique enhances the fixation of the lateral corner of the RC interval, which is beneficial for increasing the coverage area of the RC in the footprint area and promoting the healing of the cable.

The technique currently has the following characteristics: First, the double‐row suture bridge is used to repair the RC while completing biceps tenodesis and repairing the lateral corner of the RC interval, increasing the coverage area of the RC footprint and promoting cable healing. Second, the LHBT is fully preserved, which can continue to exert the biomechanical effect of LHBT in pressing the humeral head and stabilizing the shoulder.^13^ Third, the sutures of the first‐row anterior anchor simultaneously repair the supraspinatus and the lateral corner of the RC interval and complete biceps tenodesis, reducing the number of anchors and saving medical costs.

Encouraged by previous technological successes, we believe that the hybridge technique described here will be applicable to medium to large superior cuff tea with LHBT lesion, effectively completing surgeries with less anchors and costs, saving medical costs.

## DISCLOSURES

The authors (F.D., M.X.) declare that they have no known competing financial interests or personal relationships that could have appeared to influence the work reported in this article.

## References

[1] Diplock B , Hing W , Marks D . The long head of biceps at the shoulder: A scoping review. BMC Musculoskelet Disord. 2023;24:232. 36978047 10.1186/s12891-023-06346-5 PMC10044783

[2] Lalehzarian SP , Agarwalla A , Liu JN . Management of proximal biceps tendon pathology. World J Orthop. 2022;13:36‐57. 35096535 10.5312/wjo.v13.i1.36 PMC8771414

[3] Watson ST , Robbins CB , Bedi A , et al. Comparison of outcomes 1 year after rotator cuff repair with and without concomitant biceps surgery. Arthroscopy. 2017;33:1928‐1936. 28822640 10.1016/j.arthro.2017.05.009

[4] Vestermark GL , Van Doren BA , Connor PM , et al. The prevalence of rotator cuff pathology in the setting of acute proximal biceps tendon rupture. J Shoulder Elbow Surg. 2018;27:1258‐1262. 29478942 10.1016/j.jse.2018.01.006

[5] Chen CH , Hsu KY , Chen WJ , et al. Incidence and severity of biceps long head tendon lesion in patients with complete rotator cuff tears. J Trauma. 2005;58:1189‐1193. 15995469 10.1097/01.ta.0000170052.84544.34

[6] Zalneraitis BH , Milam BP , Turner EK , et al. Biceps squeeze tenotomy: Technique to improve efficiency of arthroscopic biceps tenotomy. Arthrosc Tech. 2020;9:e1851‐e1853. 33294351 10.1016/j.eats.2020.08.009 PMC7695749

[7] Martinho T , Zbinden J , Ono Y , et al. Long head of the biceps pediculated autograft augmentation of arthroscopic subscapularis repair. Arthrosc Tech. 2023;12:e1391‐e1398. 37654870 10.1016/j.eats.2023.04.008 PMC10466290

[8] Martinel V , Nourissat G , Barth J , et al. The hybridge technique: A combined technique of suture bridge and tension band for an arthroscopic eco‐responsible rotator cuff repair. Arthrosc Tech. 2022;11:e2337‐e2345. 36632402 10.1016/j.eats.2022.08.025 PMC9827004

[9] Moorthy V , Tan AHC . Should long head of biceps tenodesis or tenotomy be routinely performed in arthroscopic rotator cuff repairs? J Orthop. 2020;21:161‐165. 32255998 10.1016/j.jor.2020.03.033 PMC7114602

[10] Takahashi N , Sugaya H , Matsumoto M , et al. Progression of degenerative changes of the biceps tendon after successful rotator cuff repair. J Shoulder Elbow Surg. 2016;26:424‐429. 27914841 10.1016/j.jse.2016.09.052

[11] Takahashi N , Sugaya H , Matsuki K , et al. Hypertrophy of the extra‐articular tendon of the long head of biceps correlates with the location and size of a rotator cuff tear. Bone Joint J. 2017;99‐B:806‐811. 10.1302/0301-620X.99B6.BJJ-2016-0885.R1 28566401

[12] Zhang C , Yang G , Li T , et al. Biceps tenodesis better improves the shoulder function compared with tenotomy for long head of the biceps tendon lesions: A meta‐analysis of randomised controlled trials. J Clin Med. 2023;12:1754. 36902540 10.3390/jcm12051754 PMC10003204

[13] Erickson BJ , Basques BA , Griffin JW , et al. The effect of concomitant biceps tenodesis on reoperation rates after rotator cuff repair: A review of a large private‐payer database from 2007 to 2014. Arthroscopy. 2017;33:1301‐1307.e1. 28336230 10.1016/j.arthro.2017.01.030

